# Supplementary material for: Modeling eye gaze velocity trajectories using GANs with spectral loss for enhanced fidelity
Source: Sci Rep. 2025 Jun 6;15:19929. doi: 10.1038/s41598-025-05286-5 (PMC12144148; doi:10.1038/s41598-025-05286-5)
Supplement: Supplementary file 1 — Supplementary Information. [file 41598_2025_5286_MOESM1_ESM.pdf]

Supplementary Information: Modeling Eye Gaze Velocity Trajectories using GANs with Spectral Loss for Enhanced Fidelity

Shailendra Bhandari[1,2,3,*], **Pedro Lencastre**[1,2,3], **Rujeena Mathema**[1,2], **Alexander Szorkovszky**[4], **Anis Yazidi**[1,2,3], **and Pedro G. Lind**[1,2,3,4]

[1]Department of Computer Science, OsloMet – Oslo Metropolitan University, P.O. Box 4 St. Olavs plass, N-0130 Oslo, Norway
[2]OsloMet Artificial Intelligence Lab, Pilestredet 52, N-0166 Oslo, Norway
[3]*NordSTAR* – Nordic Center for Sustainable and Trustworthy AI Research, Pilestredet 52, N-0166 Oslo, Norway
[4]Simula Research Laboratory, Numerical Analysis and Scientific Computing, Oslo, 0164, Norway
[*]shailendra.bhandari@oslomet.no

ABSTRACT

Accurate modeling of eye gaze dynamics is essential for advancement in human-computer interaction, neurological diagnostics, and cognitive research. Traditional generative models like Markov models often fail to capture the complex temporal dependencies and distributional nuance inherent in eye gaze trajectories data. This study introduces a Generative Adversarial Network (GAN) framework employing Long Short-Term Memory (LSTM) and Convolutional Neural Network (CNN) generators and discriminators to generate high-fidelity synthetic eye gaze velocity trajectories. We conducted a comprehensive evaluation of four GAN architectures: CNN-CNN, LSTM-CNN, CNN-LSTM, and LSTM-LSTM—trained under two conditions: using only adversarial loss (L_G) and using a weighted combination of adversarial and spectral losses. Our findings reveal that the LSTM-CNN architecture trained with this new loss function exhibits the closest alignment to the real data distribution, effectively capturing both the distribution tails and the intricate temporal dependencies. The inclusion of spectral regularization significantly enhances the GANs' ability to replicate the spectral characteristics of eye gaze movements, leading to a more stable learning process and improved data fidelity. Comparative analysis with an HMM optimized to four hidden states further highlights the advantages of the LSTM-CNN GAN. Statistical metrics show that the HMM-generated data significantly diverges from the real data in terms of mean, standard deviation, skewness, and kurtosis. In contrast, the LSTM-CNN model closely matches the real data across these statistics, affirming its capacity to model the complexity of eye gaze dynamics effectively. These results position the spectrally regularized LSTM-CNN GAN as a robust tool for generating synthetic eye gaze velocity data with high fidelity. Its ability to accurately replicate both the distributional and temporal properties of real data holds significant potential for applications in simulation environments, training systems, and the development of advanced eye-tracking technologies, ultimately contributing to more naturalistic and responsive human-computer interactions.

Keywords: Generative Adversarial Networks, Stochastic Processes, Hidden Markov Models, Eye-gaze trajectories

Supplementary Information 1 Mathematical Foundation for Markov and Hidden Markov Models

A sequence of random variables $\{X_n\}_{n\geq 0}$ with values in a set E is known as a discrete-time stochastic process with state space E. In this context, the state space is assumed to be countable, and its elements are denoted by $i, j, k, \ldots$. If $X_n = i$, we say that the process is in state i at time n, or that it visits state i at time n.

Definition: the Markov property: Let $\{X_n\}_{n\geq 0}$ represent a discrete-time stochastic process with a countable state space E. If for any integer $n \geq 0$ and for all states $i_0, i_1, \ldots, i_{n-1}, i, j$,

$$P(X_{n+1} = j \mid X_n = i, X_{n-1} = i_{n-1}, \ldots, X_0 = i_0) = P(X_{n+1} = j \mid X_n = i), \tag{1}$$

whenever both sides are well-defined, this stochastic process is referred to as a Markov chain[1]. If, in addition, the right-hand side of this expression is independent of n, the chain is called a *homogeneous Markov chain (HMC)*.

The matrix $P = \{p_{ij}\}_{i,j\in E}$, where

$$p_{ij} = P(X_{n+1} = j \mid X_n = i), \tag{2}$$

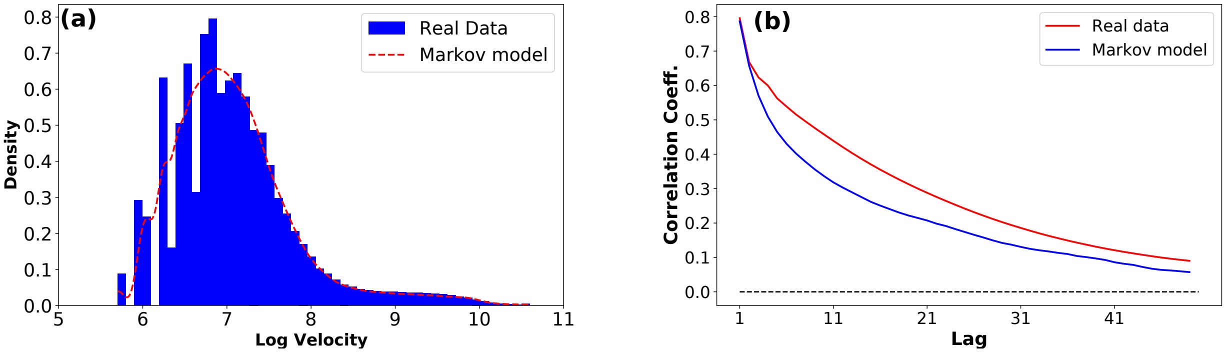

Supplementary Figure 1. (a) Histogram of log-transformed velocities in real data with the density estimation from a Markov model, illustrating the similarity in velocity distributions. The real data (blue bars) aligns closely with the Markov model's Kernel Density Estimation (KDE) curve (dashed red line), suggesting that the synthetic data captures key distributional features. (b) Autocorrelation plots of real (red line) and synthetic (blue line) data over varying lag intervals, show a faster decrease in correlation for the synthetic data compared to real data. This indicates that while the synthetic data replicates short-term dependencies, it may diverge in capturing long-term dependencies observed in the real data.

is known as the *transition matrix* of the HMC. As its entries are probabilities, and since a transition from any state i must lead to some state, it follows that

$$p_{ij} \geq 0, \quad \sum_{k \in E} p_{ik} = 1 \tag{3}$$

for all states i, j. A matrix P indexed by E and satisfying these properties is called a *stochastic matrix*. For a k-th order Markov process, the conditional probability can be estimated via Kernel Density Estimation (KDE) as:

$$P(X_n \mid X_{n-1}^{n-k}) = \frac{f(X_n, X_{n-1}^{n-k})}{f(X_{n-1}^{n-k})}, \tag{4}$$

where $X_{n-1}^{n-k} = \{X_{n-1}, X_{n-2}, \ldots, X_{n-k}\}$ and the joint probability density function $f(\cdot)$ is estimated by:

$$f(X_n, X_{n-1}^{n-k}) = \frac{1}{(N-k)h^{k+1}} \sum_{i=k+1}^{N} \prod_{j=0}^{k} K\left(\frac{X_{n-j} - X_{i-j}}{h}\right), \tag{5}$$

where $K(u)$ is the Gaussian kernel function:

$$K(u) = \frac{1}{\sqrt{2\pi}} \exp\left(-\frac{u^2}{2}\right), \tag{6}$$

and the bandwidth h is calculated using Silverman's rule[2]:

$$h = 1.06\hat{\sigma}N^{-1/(k+4)}, \tag{7}$$

where $\hat{\sigma}$ represents the standard deviation of the data.

To evaluate the effectiveness of the Markov model in capturing the statistical properties of eye-gaze velocities, we analyze the real and the generated data distributions and their autocorrelation. Figure 1 presents these comparisons in detail. Fig. 1 (a), displays the histogram of log-transformed velocities from the real data alongside the velocity eye gaze trajectories in the log scale derived from the Markov model. The close alignment between the blue bars (real data) and the dashed red line (Markov model) indicates that the synthetic data generated by the Markov model successfully captures the key distributional features of the real data. Fig. 1 (b) shows the autocorrelation plots of both real (red line) and synthetic (blue line) data over varying lag intervals. The generated data exhibits a faster decrease in autocorrelation compared to the real data, suggesting that while the Markov model replicates the short-term dependencies effectively, it may not fully capture the long-term dependencies present in the real data. This observation highlights a limitation of the Markov model in modeling temporal dependencies over longer time scales in eye-gaze velocity trajectories.

Hidden Markov Models (HMMs) are powerful statistical tools for modeling sequential data where the system being modeled is assumed to be a Markov process with unobserved (hidden) states[3]. They are beneficial in scenarios where we can observe a sequence of emissions (observable events) probabilistically dependent on a sequence of hidden states that are not directly observable. Several components formally define an HMM. First, the hidden states $S = \{s_1, s_2, \dots, s_N\}$, where N is the number of hidden states, each representing a distinct mode of the system, such as different types of eye movements. Next, the observations $O = \{o_1, o_2, \dots, o_T\}$, where T is the length of the observation sequence, with each o_t representing the observed eye-gaze velocity at time t. The initial state distribution is denoted as $\pi = \{\pi_i\}$, where $\pi_i = P(q_1 = s_i)$ is the probability that the Markov chain starts in the state s_i. The state transition probability matrix is given by $A = [a_{ij}]$, where $a_{ij} = P(q_{t+1} = s_j \mid q_t = s_i)$ represents the probability of transitioning from state s_i to state s_j. Finally, the emission probability distribution is represented as $B = \{b_j(o_t)\}$, where $b_j(o_t) = P(o_t \mid q_t = s_j)$ is the probability of observing o_t given that the system is in state s_j at time t. The complete parameter set of the HMM is denoted as $\lambda = (A, B, \pi)$. The joint probability of observing a sequence O and a particular state sequence $Q = \{q_1, q_2, \dots, q_T\}$ is expressed as:

$$P(O, Q \mid \lambda) = \pi_{q_1} b_{q_1}(o_1) \prod_{t=2}^{T} a_{q_{t-1} q_t} b_{q_t}(o_t), \tag{8}$$

where ($\lambda = \pi, A, B$) represents the parameters of the HMM. Since the states Q are hidden, the focus shifts to computing the likelihood of the observations O, which is given by:

$$P(O \mid \lambda) = \sum_{Q} P(O, Q \mid \lambda). \tag{9}$$

However, directly computing this sum is computationally infeasible for large T, as it involves summing over N^T possible state sequences. To overcome this challenge, efficient algorithms like the Forward-Backward algorithm[3] are employed. We employed the Baum-Welch algorithm, a specialized instance of the Expectation-Maximization (EM) algorithm tailored for HMMs, to estimate the model parameters $\lambda = (\pi, A, B)$. This algorithm iteratively refines the estimates of the initial state probabilities π, the state transition probabilities A, and the emission probabilities B to maximize the likelihood of the observed data[4]. In the initialization phase, we start with initial guesses for π, A, and B. The algorithm then proceeds through iterative Expectation and Maximization steps until convergence. The expectation step occupancies and expected state transition counts are computed using the forward-backward procedure. The forward probabilities $\alpha_t(i)$ and backward probabilities $\beta_t(i)$ are calculated recursively to evaluate the likelihood of partial observation sequences[5].

$$\alpha_1(i) = \pi_i b_i(o_1), \quad \alpha_{t+1}(j) = \left(\sum_{i=1}^{N} \alpha_t(i) a_{ij} \right) b_j(o_{t+1}), \tag{10}$$

where $\alpha_t(i)$ is the probability of observing the partial sequence $o_1, o_2, \dots, o_t$ and being in state s_i at time t.

$$\beta_T(i) = 1, \quad \beta_t(i) = \sum_{j=1}^{N} a_{ij} b_j(o_{t+1}) \beta_{t+1}(j), b_j(o_{t+1}), \tag{11}$$

where $\beta_t(i)$ is the probability of observing the partial sequence $o_{t+1}, o_{t+2}, \dots, o_T$ given that the state at time t is s_i. Using these probabilities, we calculate the expected state occupancy $\gamma_t(i)$ and the expected state transitions $\xi_t(i, j)$:

$$\gamma_t(i) = \frac{\alpha_t(i) \beta_t(i)}{\sum_{k=1}^{N} \alpha_t(k) \beta_t(k)}, \tag{12}$$

which represents the probability of being in state s_i at time t given the observed sequence.

$$\xi_t(i, j) = \frac{\alpha_t(i) a_{ij} b_j(o_{t+1}) \beta_{t+1}(j)}{\sum_{i=1}^{N} \sum_{j=1}^{N} \alpha_t(i) a_{ij} b_j(o_{t+1}) \beta_{t+1}(j)}, \tag{13}$$

which represents the probability of transitioning from state s_i at time t to state s_j at time $t+1$ given the observed sequence. We update the parameters to maximize the expected log-likelihood calculated in the E-step: $\pi_i = \gamma_1(i)$, ensuring that the estimated initial state probabilities reflect the expected occupancy at time $t = 1$.

$$a_{ij} = \frac{\sum_{t=1}^{T-1} \xi_t(i, j)}{\sum_{t=1}^{T-1} \gamma_t(i)}, \tag{14}$$

where the numerator is the expected number of transitions from state s_i to s_j, and the denominator is the expected number of transitions from state s_i. For a Gaussian emission model with mean μ_i and variance σ_i^2, the parameters are updated as:

$$\mu_i = \frac{\sum_{t=1}^{T} \gamma_t(i)\, o_t}{\sum_{t=1}^{T} \gamma_t(i)}, \quad \text{and} \quad \sigma_i^2 = \frac{\sum_{t=1}^{T} \gamma_t(i)\, (o_t - \mu_i)^2}{\sum_{t=1}^{T} \gamma_t(i)}, \tag{15}$$

where o_t is the observed value at time t. These expectation and maximization steps are iterated until convergence criteria are met, typically when the increase in log-likelihood between iterations falls below a predefined threshold or after reaching a maximum number of iterations. This iterative process ensures that the parameter estimates progressively improve, leading to a model that effectively captures the underlying dynamics of the eye gaze velocity trajectories. By employing the Baum-Welch algorithm, we leveraged a robust statistical framework for parameter estimation in HMMs, which is well-suited for modeling time series data with hidden structures[3]. This approach allowed us to systematically infer the hidden states and optimize the model parameters based on the observed data, facilitating a comprehensive analysis of eye movement behaviors.

References

1. Brémaud, P. *Discrete-Time Markov Chains*, 63–109 (Springer International Publishing, Cham, 2020).

2. Silverman, B. *Density Estimation for Statistics and Data Analysis* (Routledge, New York, NY, USA, 1998), 1st edn.

3. Rabiner, L. A tutorial on hidden markov models and selected applications in speech recognition. *Proc. IEEE* **77**, 257–286, DOI: 10.1109/5.18626 (1989).

4. Baum, L. E., Petrie, T., Soules, G. & Weiss, N. A maximization technique occurring in the statistical analysis of probabilistic functions of markov chains. *The Annals Math. Stat.* **41**, 164–171, DOI: 10.1214/aoms/1177697196 (1970).

5. Yu, S.-Z. Chapter 8 - variants of hsmms. In Yu, S.-Z. (ed.) *Hidden Semi-Markov Models*, 143–161, DOI: https://doi.org/10.1016/B978-0-12-802767-7.00008-5 (Elsevier, Boston, 2016).
